# Supplementary material for: Single Cell Transcriptomic and Chromatin Profiles Suggest Layer Vb Is the Only Layer With Shared Excitatory Cell Types in the Medial and Lateral Entorhinal Cortex
Source: Front Neural Circuits. 2022 Jan 26;15:806154. doi: 10.3389/fncir.2021.806154 (PMC8826650; doi:10.3389/fncir.2021.806154)
Supplement: Supplementary file 14 [file Data_Sheet_1.ZIP › 13/knownResults.html]

13 - Homer Known Motif Enrichment Results


# Homer Known Motif Enrichment Results (13)

Homer *de novo* Motif Results  
Gene Ontology Enrichment Results  
Known Motif Enrichment Results (txt file)  
Total Target Sequences = 1000, Total Background Sequences = 46712

|  |  |  |  |  |  |  |  |  |  |  |  |
| --- | --- | --- | --- | --- | --- | --- | --- | --- | --- | --- | --- |
| Rank | Motif | Name | P-value | log P-pvalue | q-value (Benjamini) | # Target Sequences with Motif | % of Targets Sequences with Motif | # Background Sequences with Motif | % of Background Sequences with Motif | Motif File | SVG |
| 1 | C A T G A G T C G A C T C G T A C G A T G C A T G A C T G C A T C G A T C T A G C A T G T G A C | Mef2b(MADS)/HEK293-Mef2b.V5-ChIP-Seq(GSE67450)/Homer | 1e-52 | -1.217e+02 | 0.0000 | 338.0 | 33.80% | 6721.8 | 14.39% | motif file (matrix) | svg |
| 2 | A G C T A C G T A C T G A T G C A G T C C G T A C T G A T A C G | NF1-halfsite(CTF)/LNCaP-NF1-ChIP-Seq(Unpublished)/Homer | 1e-48 | -1.106e+02 | 0.0000 | 437.0 | 43.70% | 10618.8 | 22.73% | motif file (matrix) | svg |
| 3 | C A T G G T A C G A C T G C T A C G T A C G T A C G T A G C T A G A C T C T G A T C A G G T A C | Mef2c(MADS)/GM12878-Mef2c-ChIP-Seq(GSE32465)/Homer | 1e-42 | -9.753e+01 | 0.0000 | 219.0 | 21.90% | 3693.3 | 7.91% | motif file (matrix) | svg |
| 4 | C A T G A G T C A G C T C G T A C G A T C G A T G C A T G C A T C G A T C T G A C A T G T G A C | Mef2d(MADS)/Retina-Mef2d-ChIP-Seq(GSE61391)/Homer | 1e-36 | -8.455e+01 | 0.0000 | 120.0 | 12.00% | 1388.8 | 2.97% | motif file (matrix) | svg |
| 5 | G T A C G A C T C G T A C T G A T C G A C G T A G C T A C A G T C T G A T A C G | Mef2a(MADS)/HL1-Mef2a.biotin-ChIP-Seq(GSE21529)/Homer | 1e-35 | -8.240e+01 | 0.0000 | 192.0 | 19.20% | 3289.1 | 7.04% | motif file (matrix) | svg |
| 6 | A T G C T A C G A G C T T G C A C G T A C G A T A C G T C T G A | DLX5(Homeobox)/BasalGanglia-Dlx5-ChIP-seq(GSE124936)/Homer | 1e-34 | -7.985e+01 | 0.0000 | 304.0 | 30.40% | 6963.4 | 14.91% | motif file (matrix) | svg |
| 7 | A T C G A T G C A G T C T A G C G A C T T C G A G C T A G C A T A G C T C T G A | DLX1(Homeobox)/BasalGanglia-Dlx1-ChIP-seq(GSE124936)/Homer | 1e-32 | -7.395e+01 | 0.0000 | 432.0 | 43.20% | 12052.6 | 25.80% | motif file (matrix) | svg |
| 8 | T C G A C A G T A C T G A G C T C G T A C G T A A C G T A C G T C T G A T A G C | Dlx3(Homeobox)/Kerainocytes-Dlx3-ChIP-Seq(GSE89884)/Homer | 1e-30 | -7.099e+01 | 0.0000 | 269.0 | 26.90% | 6088.3 | 13.03% | motif file (matrix) | svg |
| 9 | A T C G A T C G A T G C G A C T T C G A C G T A G C A T A G C T C T G A T A C G | DLX2(Homeobox)/BasalGanglia-Dlx2-ChIP-seq(GSE124936)/Homer | 1e-29 | -6.748e+01 | 0.0000 | 464.0 | 46.40% | 13712.0 | 29.35% | motif file (matrix) | svg |
| 10 | T C A G C T A G A T G C G A C T T G C A C G T A A G C T A C G T C T G A T A C G | En1(Homeobox)/SUM149-EN1-ChIP-Seq(GSE120957)/Homer | 1e-26 | -6.076e+01 | 0.0000 | 503.0 | 50.30% | 15770.3 | 33.76% | motif file (matrix) | svg |
| 11 | A G C T G T C A C G T A A C G T A C G T C T G A T C A G A T G C | Lhx2(Homeobox)/HFSC-Lhx2-ChIP-Seq(GSE48068)/Homer | 1e-24 | -5.688e+01 | 0.0000 | 344.0 | 34.40% | 9472.5 | 20.28% | motif file (matrix) | svg |
| 12 | A C T G C A G T A C G T C G T A C G T A A C G T A C T G C T G A | Nkx6.1(Homeobox)/Islet-Nkx6.1-ChIP-Seq(GSE40975)/Homer | 1e-20 | -4.626e+01 | 0.0000 | 621.0 | 62.10% | 22151.4 | 47.42% | motif file (matrix) | svg |
| 13 | A T G C T A C G A G T C G A C T T G C A C T G A G A C T A C G T C T G A T C A G | LHX9(Homeobox)/Hct116-LHX9.V5-ChIP-Seq(GSE116822)/Homer | 1e-18 | -4.234e+01 | 0.0000 | 389.0 | 38.90% | 12159.8 | 26.03% | motif file (matrix) | svg |
| 14 | A G T C C G A T A C T G A T C G T G A C G C T A C A T G A T C G T G A C C G A T A C T G T A G C G T A C G T C A | Tlx?(NR)/NPC-H3K4me1-ChIP-Seq(GSE16256)/Homer | 1e-15 | -3.643e+01 | 0.0000 | 114.0 | 11.40% | 2283.5 | 4.89% | motif file (matrix) | svg |
| 15 | G A C T C T A G A T G C A G T C G T C A T A C G A T G C A T C G | HIC1(Zf)/Treg-ZBTB29-ChIP-Seq(GSE99889)/Homer | 1e-12 | -2.984e+01 | 0.0000 | 356.0 | 35.60% | 11726.7 | 25.10% | motif file (matrix) | svg |
| 16 | C G T A C G T A A G C T G A C T T G C A G T C A A C G T A G C T C T G A T C A G | Lhx3(Homeobox)/Neuron-Lhx3-ChIP-Seq(GSE31456)/Homer | 1e-11 | -2.725e+01 | 0.0000 | 433.0 | 43.30% | 15258.2 | 32.66% | motif file (matrix) | svg |
| 17 | C G T A C A T G A G T C G A C T T G C A C G T A A C G T A C G T C T G A T C A G | Lhx1(Homeobox)/EmbryoCarcinoma-Lhx1-ChIP-Seq(GSE70957)/Homer | 1e-11 | -2.697e+01 | 0.0000 | 323.0 | 32.30% | 10591.0 | 22.67% | motif file (matrix) | svg |
| 18 | A G T C G A C T C A G T A C T G C T A G T G A C G C T A A T G C G C A T A T C G C G A T A C T G G A T C G T A C G T C A C T G A | NF1(CTF)/LNCAP-NF1-ChIP-Seq(Unpublished)/Homer | 1e-10 | -2.393e+01 | 0.0000 | 87.0 | 8.70% | 1883.4 | 4.03% | motif file (matrix) | svg |
| 19 | C T A G T A C G G A T C G T A C G C T A A G C T A G C T G T C A T C G A T A G C | Nanog(Homeobox)/mES-Nanog-ChIP-Seq(GSE11724)/Homer | 1e-8 | -2.069e+01 | 0.0000 | 725.0 | 72.50% | 29668.6 | 63.51% | motif file (matrix) | svg |
| 20 | A T G C G A T C C G T A A G C T C A G T A T C G G C A T A G C T G A C T A C T G | Sox17(HMG)/Endoderm-Sox17-ChIP-Seq(GSE61475)/Homer | 1e-7 | -1.648e+01 | 0.0000 | 175.0 | 17.50% | 5496.7 | 11.77% | motif file (matrix) | svg |
| 21 | A G T C C G A T C T G A C G T A A C G T C A G T T C A G T G A C | Isl1(Homeobox)/Neuron-Isl1-ChIP-Seq(GSE31456)/Homer | 1e-6 | -1.577e+01 | 0.0000 | 399.0 | 39.90% | 15010.2 | 32.13% | motif file (matrix) | svg |
| 22 | A G T C G C A T C G T A C G T A G T A C A C G T A C T G G A T C G A T C T C G A | BMYB(HTH)/Hela-BMYB-ChIP-Seq(GSE27030)/Homer | 1e-6 | -1.543e+01 | 0.0000 | 284.0 | 28.40% | 10063.0 | 21.54% | motif file (matrix) | svg |
| 23 | C T A G T A C G G A T C G T C A T G C A A C G T T G C A G C T A T C G A T G C A | Hoxa9(Homeobox)/ChickenMSG-Hoxa9.Flag-ChIP-Seq(GSE86088)/Homer | 1e-6 | -1.492e+01 | 0.0000 | 553.0 | 55.30% | 22138.2 | 47.39% | motif file (matrix) | svg |
| 24 | A T G C A G T C G A T C C G T A A C G T A C G T A C T G A C G T A G C T G A T C | Sox2(HMG)/mES-Sox2-ChIP-Seq(GSE11431)/Homer | 1e-6 | -1.464e+01 | 0.0000 | 202.0 | 20.20% | 6740.0 | 14.43% | motif file (matrix) | svg |
| 25 | A T G C G A T C C G A T A C G T A C G T A C T G C A G T A G C T | Sox3(HMG)/NPC-Sox3-ChIP-Seq(GSE33059)/Homer | 1e-6 | -1.407e+01 | 0.0000 | 356.0 | 35.60% | 13333.2 | 28.54% | motif file (matrix) | svg |
| 26 | C T A G C T A G A G T C T C A G A C T G A C G T A C G T C T G A | MYB(HTH)/ERMYB-Myb-ChIPSeq(GSE22095)/Homer | 1e-5 | -1.345e+01 | 0.0000 | 314.0 | 31.40% | 11583.6 | 24.80% | motif file (matrix) | svg |
| 27 | C T G A T C G A C G T A A T G C C G T A C G T A C G A T C T A G T C A G G A T C | Sox15(HMG)/CPA-Sox15-ChIP-Seq(GSE62909)/Homer | 1e-5 | -1.320e+01 | 0.0000 | 239.0 | 23.90% | 8418.9 | 18.02% | motif file (matrix) | svg |
| 28 | G A C T C T A G C T A G A G T C T G C A A C T G A C G T A C G T C T A G T C A G | AMYB(HTH)/Testes-AMYB-ChIP-Seq(GSE44588)/Homer | 1e-5 | -1.219e+01 | 0.0001 | 273.0 | 27.30% | 9979.2 | 21.36% | motif file (matrix) | svg |
| 29 | A T G C A G T C G C A T A G C T A C G T T C A G C G A T A G C T G A T C A T C G | Sox10(HMG)/SciaticNerve-Sox3-ChIP-Seq(GSE35132)/Homer | 1e-5 | -1.203e+01 | 0.0001 | 329.0 | 32.90% | 12426.6 | 26.60% | motif file (matrix) | svg |
| 30 | G A C T G T A C T G C A A C G T G A T C G C T A T C G A A C G T A G T C C G T A | Pdx1(Homeobox)/Islet-Pdx1-ChIP-Seq(SRA008281)/Homer | 1e-5 | -1.195e+01 | 0.0001 | 233.0 | 23.30% | 8311.3 | 17.79% | motif file (matrix) | svg |
| 31 | A T G C G T A C C G T A A G C T G C A T T A C G A G C T A G C T A G T C A G C T | Sox6(HMG)/Myotubes-Sox6-ChIP-Seq(GSE32627)/Homer | 1e-5 | -1.153e+01 | 0.0001 | 340.0 | 34.00% | 12979.2 | 27.79% | motif file (matrix) | svg |
| 32 | A G C T C A T G G C A T G A T C T G C A C T A G G A T C A C G T | Tgif2(Homeobox)/mES-Tgif2-ChIP-Seq(GSE55404)/Homer | 1e-4 | -1.126e+01 | 0.0002 | 528.0 | 52.80% | 21535.8 | 46.10% | motif file (matrix) | svg |
| 33 | C A G T T C A G A G C T G A C T A C G T A G T C G A T C G A C T C T G A A C T G G A T C C G T A C T G A A G T C G T A C | Rfx6(HTH)/Min6b1-Rfx6.HA-ChIP-Seq(GSE62844)/Homer | 1e-4 | -1.114e+01 | 0.0002 | 186.0 | 18.60% | 6447.4 | 13.80% | motif file (matrix) | svg |
| 34 | T G A C T A G C T C A G T C G A T C G A C G T A A G T C C G T A C G T A C G A T C T A G T A C G | Sox7(HMG)/ESC-Sox7-ChIP-Seq(GSE133899)/Homer | 1e-4 | -1.012e+01 | 0.0005 | 72.0 | 7.20% | 2050.0 | 4.39% | motif file (matrix) | svg |
| 35 | C G T A A C G T A C G T A C G T A C G T A G T C A G T C C T G A A G C T A G C T | NFAT(RHD)/Jurkat-NFATC1-ChIP-Seq(Jolma\_et\_al.)/Homer | 1e-4 | -9.955e+00 | 0.0006 | 203.0 | 20.30% | 7294.8 | 15.62% | motif file (matrix) | svg |
| 36 | C T G A T C A G G T A C G C T A A C T G T G A C G C A T C A T G | SCL(bHLH)/HPC7-Scl-ChIP-Seq(GSE13511)/Homer | 1e-4 | -9.608e+00 | 0.0008 | 645.0 | 64.50% | 27348.7 | 58.55% | motif file (matrix) | svg |
| 37 | C T A G T A C G G A T C G T A C C T G A A G C T T G C A G C T A C G T A G C A T G A T C G C T A | Hoxc9(Homeobox)/Ainv15-Hoxc9-ChIP-Seq(GSE21812)/Homer | 1e-3 | -9.147e+00 | 0.0013 | 128.0 | 12.80% | 4298.4 | 9.20% | motif file (matrix) | svg |
| 38 | C T A G A T G C A T G C C G A T A C T G G A C T A T G C G C T A T G A C A G C T T A G C G C T A | PBX1(Homeobox)/MCF7-PBX1-ChIP-Seq(GSE28007)/Homer | 1e-3 | -9.046e+00 | 0.0014 | 22.0 | 2.20% | 411.0 | 0.88% | motif file (matrix) | svg |
| 39 | A G T C A G T C C G A T A C G T A C G T A C T G A C G T A G C T A G T C A G T C | Sox4(HMG)/proB-Sox4-ChIP-Seq(GSE50066)/Homer | 1e-3 | -8.709e+00 | 0.0019 | 174.0 | 17.40% | 6236.4 | 13.35% | motif file (matrix) | svg |
| 40 | T C A G A G C T A C G T A C G T G T A C G A T C C G T A C T A G C A T G G T C A C G T A T C G A | STAT4(Stat)/CD4-Stat4-ChIP-Seq(GSE22104)/Homer | 1e-3 | -8.703e+00 | 0.0019 | 190.0 | 19.00% | 6909.0 | 14.79% | motif file (matrix) | svg |
| 41 | T A C G A T G C G A C T A C T G A G C T A G T C G T C A T G C A A C G T A G T C G C T A T G C A | Pknox1(Homeobox)/ES-Prep1-ChIP-Seq(GSE63282)/Homer | 1e-3 | -8.364e+00 | 0.0025 | 47.0 | 4.70% | 1258.2 | 2.69% | motif file (matrix) | svg |
| 42 | C T G A C T A G A T C G G C A T A C T G G T A C A T G C C G T A A C T G G C T A A G T C C G T A | Tbox:Smad(T-box,MAD)/ESCd5-Smad2\_3-ChIP-Seq(GSE29422)/Homer | 1e-3 | -8.340e+00 | 0.0025 | 38.0 | 3.80% | 947.8 | 2.03% | motif file (matrix) | svg |
| 43 | A C T G C T A G T C G A C G A T C A T G G C T A A T C G C G A T G T A C G C T A A G C T G T A C | Fra1(bZIP)/BT549-Fra1-ChIP-Seq(GSE46166)/Homer | 1e-3 | -8.336e+00 | 0.0025 | 102.0 | 10.20% | 3341.2 | 7.15% | motif file (matrix) | svg |
| 44 | A G T C G A T C G C T A C G A T A C G T T A C G G C A T C T G A G A C T A C T G A G T C G C T A C T G A T C G A C A G T | Oct4:Sox17(POU,Homeobox,HMG)/F9-Sox17-ChIP-Seq(GSE44553)/Homer | 1e-3 | -8.248e+00 | 0.0026 | 43.0 | 4.30% | 1124.1 | 2.41% | motif file (matrix) | svg |
| 45 | T C G A A C G T A C G T C T G A G A T C T C A G G A C T G T C A C G T A A G C T G T C A C T A G A G C T A C G T T C G A | NFIL3(bZIP)/HepG2-NFIL3-ChIP-Seq(Encode)/Homer | 1e-3 | -8.238e+00 | 0.0026 | 138.0 | 13.80% | 4803.3 | 10.28% | motif file (matrix) | svg |
| 46 | C A T G C T A G T C G A A C G T A C T G C G T A T A G C C G A T T G A C C G T A A G C T G A T C | Fra2(bZIP)/Striatum-Fra2-ChIP-Seq(GSE43429)/Homer | 1e-3 | -8.078e+00 | 0.0030 | 84.0 | 8.40% | 2659.9 | 5.69% | motif file (matrix) | svg |
| 47 | T A G C T A G C G A C T C T A G A G C T A G T C G T C A T G C A A C G T A T G C G C T A T G C A | Pbx3(Homeobox)/GM12878-PBX3-ChIP-Seq(GSE32465)/Homer | 1e-3 | -8.065e+00 | 0.0030 | 49.0 | 4.90% | 1347.6 | 2.88% | motif file (matrix) | svg |
| 48 | G C A T A T C G C A T G G T A C G C T A A G T C T C A G T G A C G T C A T G C A | Arnt:Ahr(bHLH)/MCF7-Arnt-ChIP-Seq(Lo\_et\_al.)/Homer | 1e-3 | -7.964e+00 | 0.0032 | 104.0 | 10.40% | 3458.0 | 7.40% | motif file (matrix) | svg |
| 49 | A C T G C A T G T C G A A C G T A C T G C G T A A T C G A C G T G T A C C G T A G A C T A G T C | Fos(bZIP)/TSC-Fos-ChIP-Seq(GSE110950)/Homer | 1e-3 | -7.874e+00 | 0.0034 | 106.0 | 10.60% | 3546.8 | 7.59% | motif file (matrix) | svg |
| 50 | G T C A T G C A G C T A A G T C C G T A A C T G T G A C G C A T T C A G C A G T | Ap4(bHLH)/AML-Tfap4-ChIP-Seq(GSE45738)/Homer | 1e-3 | -7.779e+00 | 0.0037 | 200.0 | 20.00% | 7460.9 | 15.97% | motif file (matrix) | svg |
| 51 | C T A G T C G A C G A T A C T G C G T A T A C G A G C T T G A C G C T A A C G T G A T C T A G C | Fosl2(bZIP)/3T3L1-Fosl2-ChIP-Seq(GSE56872)/Homer | 1e-3 | -7.627e+00 | 0.0042 | 55.0 | 5.50% | 1594.3 | 3.41% | motif file (matrix) | svg |
| 52 | C G T A C T A G T C A G T C A G A G T C A T G C A G T C G C A T A G C T A C G T A T C G C G A T | Sox9(HMG)/Limb-SOX9-ChIP-Seq(GSE73225)/Homer | 1e-3 | -7.276e+00 | 0.0059 | 169.0 | 16.90% | 6216.5 | 13.31% | motif file (matrix) | svg |
| 53 | C G T A C T G A C T A G C G T A C G T A A G T C C G T A C A G T G C A T G T C A C G A T A C T G A C G T G C A T G A T C | PGR(NR)/EndoStromal-PGR-ChIP-Seq(GSE69539)/Homer | 1e-3 | -7.076e+00 | 0.0070 | 54.0 | 5.40% | 1596.2 | 3.42% | motif file (matrix) | svg |
| 54 | T C A G G A C T C A G T C T G A A G C T C T A G G A C T T G C A C T G A A G T C | HLF(bZIP)/HSC-HLF.Flag-ChIP-Seq(GSE69817)/Homer | 1e-3 | -7.041e+00 | 0.0071 | 178.0 | 17.80% | 6631.0 | 14.20% | motif file (matrix) | svg |
| 55 | G T A C C G T A C G T A T A C G G C A T G T A C C G T A C A T G A G T C C G T A C G T A C G A T G C A T G C A T G A C T | MafF(bZIP)/HepG2-MafF-ChIP-Seq(GSE31477)/Homer | 1e-2 | -6.886e+00 | 0.0082 | 75.0 | 7.50% | 2416.0 | 5.17% | motif file (matrix) | svg |
| 56 | C T A G T C G A A C G T A C T G C G T A A T G C A C G T G T A C C G T A A G C T G A T C G T A C | Atf3(bZIP)/GBM-ATF3-ChIP-Seq(GSE33912)/Homer | 1e-2 | -6.775e+00 | 0.0090 | 113.0 | 11.30% | 3950.3 | 8.46% | motif file (matrix) | svg |
| 57 | A G T C A C G T A C T G A G C T A C G T A C G T G T C A A G T C | Foxo1(Forkhead)/RAW-Foxo1-ChIP-Seq(Fan\_et\_al.)/Homer | 1e-2 | -6.418e+00 | 0.0126 | 324.0 | 32.40% | 13133.9 | 28.12% | motif file (matrix) | svg |
| 58 | C A T G C T A G A G C T G A C T C A T G A G T C G A T C G C T A C G A T C T A G T C A G G T A C C T G A T C G A | X-box(HTH)/NPC-H3K4me1-ChIP-Seq(GSE16256)/Homer | 1e-2 | -6.305e+00 | 0.0139 | 27.0 | 2.70% | 675.0 | 1.45% | motif file (matrix) | svg |
| 59 | T C A G G T C A G C T A A G T C C G T A A T C G T C G A T C A G C G T A A C T G A C T G A C T G | ZNF768(Zf)/Rajj-ZNF768-ChIP-Seq(GSE111879)/Homer | 1e-2 | -6.057e+00 | 0.0175 | 10.0 | 1.00% | 157.0 | 0.34% | motif file (matrix) | svg |
| 60 | A C G T A G T C A G T C C G A T A C G T A C G T A C T G A C G T A T G C G A C T A C T G T A C G | Sox21(HMG)/ESC-SOX21-ChIP-Seq(GSE110505)/Homer | 1e-2 | -5.971e+00 | 0.0187 | 354.0 | 35.40% | 14577.5 | 31.21% | motif file (matrix) | svg |
| 61 | C A G T T G C A A C G T A C T G C G T A A T C G C G A T T G A C C G T A A C G T | BATF(bZIP)/Th17-BATF-ChIP-Seq(GSE39756)/Homer | 1e-2 | -5.882e+00 | 0.0201 | 112.0 | 11.20% | 4019.5 | 8.60% | motif file (matrix) | svg |
| 62 | C T G A T C A G A G T C C G T A A T C G A T G C C G A T A C T G A G T C G A C T A T C G A G T C | MyoD(bHLH)/Myotube-MyoD-ChIP-Seq(GSE21614)/Homer | 1e-2 | -5.868e+00 | 0.0201 | 118.0 | 11.80% | 4270.6 | 9.14% | motif file (matrix) | svg |
| 63 | T C G A A G T C C G T A A T C G A T G C C G A T A C T G A G T C A G C T A C T G | Tcf12(bHLH)/GM12878-Tcf12-ChIP-Seq(GSE32465)/Homer | 1e-2 | -5.812e+00 | 0.0209 | 140.0 | 14.00% | 5201.0 | 11.13% | motif file (matrix) | svg |
| 64 | T A G C G T A C C G T A C T A G A C T G T G C A C G T A A T G C C G T A A T C G | AR-halfsite(NR)/LNCaP-AR-ChIP-Seq(GSE27824)/Homer | 1e-2 | -5.743e+00 | 0.0220 | 567.0 | 56.70% | 24454.9 | 52.35% | motif file (matrix) | svg |
| 65 | T A C G T C G A G A C T A C T G C T G A A G T C T C A G G A C T T G A C C T G A | Atf1(bZIP)/K562-ATF1-ChIP-Seq(GSE31477)/Homer | 1e-2 | -5.656e+00 | 0.0237 | 104.0 | 10.40% | 3717.3 | 7.96% | motif file (matrix) | svg |
| 66 | A T G C T C G A A G T C A G C T A C G T G T A C A G T C G C T A C T A G C A T G G T C A C T G A T C A G A G T C | Stat3+il21(Stat)/CD4-Stat3-ChIP-Seq(GSE19198)/Homer | 1e-2 | -5.640e+00 | 0.0237 | 125.0 | 12.50% | 4594.8 | 9.84% | motif file (matrix) | svg |
| 67 | T C A G A C G T A G T C T C G A A G T C T C A G G C A T C T A G C T A G A G C T | Usf2(bHLH)/C2C12-Usf2-ChIP-Seq(GSE36030)/Homer | 1e-2 | -5.636e+00 | 0.0237 | 52.0 | 5.20% | 1633.4 | 3.50% | motif file (matrix) | svg |
| 68 | A C G T T C G A T C G A A G T C G T C A T A C G A T G C A C G T A C T G A G C T | Myf5(bHLH)/GM-Myf5-ChIP-Seq(GSE24852)/Homer | 1e-2 | -5.468e+00 | 0.0273 | 109.0 | 10.90% | 3949.6 | 8.46% | motif file (matrix) | svg |
| 69 | T G C A G C A T C G A T C G T A C A G T A C T G G T A C C G T A C T G A A G C T G T C A A C T G C T A G G T C A C G A T A C T G G T A C T G C A C G T A A G C T | CEBP:CEBP(bZIP)/MEF-Chop-ChIP-Seq(GSE35681)/Homer | 1e-2 | -5.443e+00 | 0.0276 | 38.0 | 3.80% | 1118.4 | 2.39% | motif file (matrix) | svg |
| 70 | T G C A C G T A G T C A A G C T A G T C G C T A T A G C C G A T C T A G G A T C | Gfi1b(Zf)/HPC7-Gfi1b-ChIP-Seq(GSE22178)/Homer | 1e-2 | -5.321e+00 | 0.0307 | 125.0 | 12.50% | 4641.0 | 9.94% | motif file (matrix) | svg |
| 71 | C T A G A C G T A G T C C G T A A C T G A G T C G C A T A C T G G C A T A G T C G A C T G A T C G C A T A G T C A G C T | ZNF317(Zf)/HEK293-ZNF317.GFP-ChIP-Seq(GSE58341)/Homer | 1e-2 | -5.314e+00 | 0.0307 | 20.0 | 2.00% | 485.4 | 1.04% | motif file (matrix) | svg |
| 72 | T G A C C T G A C T A G C T G A C G T A A G T C C T G A A C G T G C A T T A G C G C A T A T C G G A C T G A C T G A T C | GRE(NR),IR3/RAW264.7-GRE-ChIP-Seq(Unpublished)/Homer | 1e-2 | -5.307e+00 | 0.0307 | 45.0 | 4.50% | 1392.1 | 2.98% | motif file (matrix) | svg |
| 73 | G A C T C G A T C T G A G T C A G A C T C G A T T C G A C G T A G C T A G C T A T G A C G T A C C G T A A C T G T G C A C G A T A C T G A C G T | Pitx1:Ebox(Homeobox,bHLH)/Hindlimb-Pitx1-ChIP-Seq(GSE41591)/Homer | 1e-2 | -5.241e+00 | 0.0319 | 47.0 | 4.70% | 1474.7 | 3.16% | motif file (matrix) | svg |
| 74 | G A C T G C A T C T A G C G A T G A T C T C G A C A T G G A T C | Tgif1(Homeobox)/mES-Tgif1-ChIP-Seq(GSE55404)/Homer | 1e-2 | -4.976e+00 | 0.0410 | 482.0 | 48.20% | 20681.2 | 44.27% | motif file (matrix) | svg |
| 75 | T C G A A C G T C A T G G C T A T A G C C G A T G T A C G C T A A C G T A T G C | AP-1(bZIP)/ThioMac-PU.1-ChIP-Seq(GSE21512)/Homer | 1e-2 | -4.875e+00 | 0.0448 | 118.0 | 11.80% | 4411.9 | 9.45% | motif file (matrix) | svg |
| 76 | C G A T C T A G T C G A A G C T C G A T C T G A C G T A A G C T A C T G C T A G A T G C G A T C | Hoxb4(Homeobox)/ES-Hoxb4-ChIP-Seq(GSE34014)/Homer | 1e-2 | -4.850e+00 | 0.0453 | 46.0 | 4.60% | 1469.5 | 3.15% | motif file (matrix) | svg |
| 77 | C T A G T C G A A C G T A C T G C G T A T A G C C G A T G T A C C G T A A G C T G A T C G T A C | Jun-AP1(bZIP)/K562-cJun-ChIP-Seq(GSE31477)/Homer | 1e-2 | -4.698e+00 | 0.0521 | 37.0 | 3.70% | 1136.5 | 2.43% | motif file (matrix) | svg |
| 78 | A G T C C T G A A G T C C G A T C A G T G A T C A T G C A C T G A T C G G A C T | Fli1(ETS)/CD8-FLI-ChIP-Seq(GSE20898)/Homer | 1e-2 | -4.677e+00 | 0.0525 | 161.0 | 16.10% | 6286.5 | 13.46% | motif file (matrix) | svg |
| 79 | T C G A A C G T A C T G C T G A A G T C T C A G A G C T G T A C C G T A A G C T G A T C T C G A | JunD(bZIP)/K562-JunD-ChIP-Seq/Homer | 1e-2 | -4.616e+00 | 0.0551 | 16.0 | 1.60% | 383.6 | 0.82% | motif file (matrix) | svg |
| 80 | A C T G T C A G A G C T G A C T C A T G A G T C A G T C G C T A C G A T C T A G T C A G G T A C C T G A T C G A | Rfx1(HTH)/NPC-H3K4me1-ChIP-Seq(GSE16256)/Homer | 1e-2 | -4.609e+00 | 0.0551 | 37.0 | 3.70% | 1143.9 | 2.45% | motif file (matrix) | svg |
| 81 | C T A G T A G C A G T C G T C A C T G A A C G T C G T A C G T A C G T A G C T A | Hoxd13(Homeobox)/ChickenMSG-Hoxd13.Flag-ChIP-Seq(GSE86088)/Homer | 1e-2 | -4.609e+00 | 0.0551 | 347.0 | 34.70% | 14580.1 | 31.21% | motif file (matrix) | svg |
